# Building implementation capacity (BIC): a longitudinal mixed methods evaluation of a team intervention

**DOI:** 10.1186/s12913-019-4086-1

**Published:** 2019-05-07

**Authors:** Rebecca Mosson, Hanna Augustsson, Annika Bäck, Mårten Åhström, Ulrica von Thiele Schwarz, Anne Richter, Malin Gunnarsson, Henna Hasson

**Affiliations:** 1 Department of Learning, Informatics, Management and Ethics, Medical Management Centre, Karolinska Institutet, Tomtebodavägen 18a, 17177 Stockholm, Sweden; 2 Center for Epidemiology and Community Medicine, Stockholm County Council, Stockholm, Sweden; 3 School of Health, Care and Social Welfare, Mälardalen University, Box 883, 721 23 Västerås, Sweden

**Keywords:** Skills training, Tailored implementation, Learning, Work groups, Managers

## Abstract

**Background:**

Managers and professionals in health and social care are required to implement evidence-based methods. Despite this, they generally lack training in implementation. In clinical settings, implementation is often a team effort, so it calls for team training. The aim of this study was to evaluate the effects of the Building Implementation Capacity (BIC) intervention that targets teams of professionals, including their managers.

**Methods:**

A non-randomized design was used, with two intervention cases (each consisting of two groups). The longitudinal, mixed-methods evaluation included pre–post and workshop-evaluation questionnaires, and interviews following Kirkpatrick’s four-level evaluation framework. The intervention was delivered in five workshops, using a systematic implementation method with exercises and practical working materials. To improve transfer of training, the teams’ managers were included. Practical experiences were combined with theoretical knowledge, social interactions, reflections, and peer support.

**Results:**

Overall, the participants were satisfied with the intervention (first level), and all groups increased their self-rated implementation knowledge (second level). The qualitative results indicated that most participants applied what they had learned by enacting new implementation behaviors (third level). However, they only partially applied the implementation method, as they did not use the planned systematic approach. A few changes in organizational results occurred (fourth level).

**Conclusions:**

The intervention had positive effects with regard to the first two levels of the evaluation model; that is, the participants were satisfied with the intervention and improved their knowledge and skills. Some positive changes also occurred on the third level (behaviors) and fourth level (organizational results), but these were not as clear as the results for the first two levels. This highlights the fact that further optimization is needed to improve transfer of training when building teams’ implementation capacity. In addition to considering the design of such interventions, the organizational context and the participants’ characteristics may also need to be considered to maximize the chances that the learned skills will be successfully transferred to behaviors.

**Electronic supplementary material:**

The online version of this article (10.1186/s12913-019-4086-1) contains supplementary material, which is available to authorized users.

## Background

Implementation science has made great contributions to the knowledge about which strategies increase the use of evidence-based methods in health care [1–4]. For instance, having common goals, understanding the hindrances and facilitators, and continuously measuring process and outcomes are all important activities in implementation. However, these activities require specific knowledge from beyond health care managers’ and professionals’ training; therefore, they often lack skills for implementing evidence-based methods [5–7]. Consequently, implementation often occurs without careful planning or a structured approach, which results in the use of strategies that do not match organizational needs or that do not include evaluations of the implementation [7, 8]. This emphasizes the need to train managers and professionals in the skills they need to provide more effective implementations and to thus improve care quality and service efficiency.

Most implementation trainings have targeted researchers or doctoral and master’s-level students [9–15]. Some trainings have targeted specific groups (e.g., health care managers [16–18]), whereas others have targeted individual professionals through university courses, webinars [11], or a combination of workshops and webinars [15]. However, the implementation of evidence-based methods typically involves several individuals [19] who depend on the same immediate manager [20–22]. Hence, working together as a team is important to ensure that all members understand their roles in the implementation [8]. Consequently, implementation training should target teams rather than individuals [8]. Targeting teams allows for consideration of each team member’s unique role and supports the team’s pursuit of implementation as a collective effort (in addition to individual skill training). Furthermore, team training can create common implementation goals and work processes [8]. Team training can also help team members to efficiently identify local hindrances through their unique knowledge about the local context [23, 24]. In fact, team training has proven more effective than individual training in technology implementation [25]. However, to the authors’ knowledge, there are no prior evaluations of team trainings on the implementation of evidence-based methods.

For a team training to be effective, understanding how teams function and learn is crucial. Team learning is a social process [19] in which members acquire, share, and combine knowledge through shared experiences [26]. Adults tend to learn by reflecting on concrete experiences from everyday practice [27, 28], and adult learning happens in cycles [27]. For instance, after encountering a new experience (e.g., an attempt to implement a new routine), a learner creates meaning for that experience. Through reflection, the experience-specific learning becomes more general, which can result in ideas for new approaches that the learner then applies in practice, leading to new reflections and learning [27]. When applying this cycle to team learning, group discussions can accelerate learning by facilitating the reflection phase [19, 29]. Group discussions can also help members to contribute their unique perspectives on the phenomena at hand [19]. This is particularly true for diverse groups such as multiprofessional groups. Taken together, this evidence suggests that team reflections and discussions regarding concrete, practical workplace experiences are important in team training.

A challenge in any training is the risk that participants will not use the learned skills in their work [30–32]. The literature on training transfer suggests that the use of learned skills in practice is influenced by three factors: the participants’ characteristics, their organizational context, and the training intervention design [30, 32]. The individuals’ characteristics include their motivations, abilities, personalities, and existing skills. The post-training organizational context includes the organizational climate, management support and feedback, and the opportunities to put the skills into practice. The intervention design consists of the training’s objectives, content, and pedagogical methods. For those who design trainings, the participants’ individual characteristics are difficult to manipulate, particularly when there is no way to influence who will participate. Instead, to improve the chances of training transfer, intervention developers can focus on the intervention design and, to some extent, on the organizational context [30]. One way to make the organizational context more receptive to the transfer of learned skills into practical behaviors is to include managers in the training. Managers have unique opportunities to create prerequisites for their subordinates to use the acquired skills in the workplace. Another option is to train an entire organization so as to develop a common mental model of implementation and to secure the support of significant organizational stakeholders such as senior managers [33, 34]. With regard to the intervention design, training developers can choose the most suitable pedagogical activities for the objectives. For instance, when the learning goal is to perform an activity (rather than to simply learn how to do it), a suitable pedagogical technique is practice (e.g., role play). Other pedagogical methods—for instance, group work (e.g., group reflections), peer learning (e.g., exchange experiences), and testing changes in the workplace—can also be effective [30, 35].

Altogether, the evidence suggests that managers and professionals in health and social care need training to enable effective implementation. Team training may be an effective way to improve managers’ and professionals’ skills and to optimize the transfer of the learned skills into practice. Therefore, this study’s aim is to evaluate the effects of the Building Implementation Capacity (BIC) intervention that targets teams of professionals, including their managers.

## Methods

A non-randomized intervention study with two intervention cases (each consisting of two groups) was conducted in 2016 and 2017. The longitudinal, mixed-methods evaluation included pre–post and workshop evaluation questionnaires, as well as interviews that were performed following Kirkpatrick’s four-level evaluation framework [36]. The study was carried out in the Stockholm region of Sweden, which is one of Sweden’s largest health care providers serving a population of two million, and is responsible for all health care provided by the regional authority. This includes primary care, acute hospital care and psychiatric rehabilitation. The study was a collaboration between academic researchers, local R&D unit and local health care and social care organizations. The researchers’ role was to design the intervention and the evaluation, and the R&D unit that employed some of the researchers that were responsible for conducting the BIC intervention.

### Participants

The target population comprised teams of health care and social care work units in the region. Each team consisted of professionals’ that provide direct services to patients and clients and their immediate manager. The recruitment process was somewhat distinct for Case 1 and Case 2.

*Case 1*: In 2014, a local organization that provides elderly care and disability care approached the R&D unit to request help in building its professionals’ implementation capacity. The last author met with this organization’s senior managers on six occasions during 2014 and 2015 to discuss their needs and objectives. The senior managers decided that all work units should participate in the intervention, and an offer to participate was e-mailed to all 26 unit managers. In total, 20 units decided to participate with one team. For logistical reasons, the teams were divided into two groups that would participate in spring 2016 (Intervention group 1) or in autumn 2016 (Intervention group 2). The allocation of the teams was inspired by a stepped-wedge design that randomly allocated them into participating in Intervention group 1 or Intervention group 2 with the ambition that each team could act as their own control. However, ten of the participating teams expressed specific wishes in regards to when to participate in the intervention (e.g., a preference to participate in the spring due to organizational changes occurring in autumn). These wishes were considered for pragmatic reasons; however, as a result, ten teams selected when to participate in the intervention, whereas the other teams were randomized according to scope of practice of each unit. Two teams also wished to change intervention group after the randomization of teams was performed. Thus, the stepped wedge approach was not fully adhered to when allocating the teams into intervention groups.

*Case 2*: An invitation to participate in the intervention was distributed to health care and social care organizations in the region through emails to the division managers and to the managers and other professionals on the R&D unit’s mailing list (approximately 600 individuals), as well as a post on the unit’s web site. The recruitment was conducted between September and November 2016 for Intervention group 3 and between May and August 2016 for Intervention group 4. The exact number of organizations that were reached by the invitation cannot be determined. In Case 2, 19 units, i.e., teams of professionals and the immediate manager, signed up to participate in the intervention. All units that signed up for participation were included in the intervention, since they met the inclusion criteria mentioned below. However, four units withdrew their participation before the intervention commenced. This resulted in 15 units consisting of teams both within the same organization and teams from different organizations.

All participating units, regardless of case, were asked to provide 1) a specific implementation to actively work on during the intervention and 2) the team, i.e., the manager and key professionals, who would participate in the intervention.

### Intervention cases

Based on the recruitment process, the teams in the two cases differed with regard to the intervention’s embeddedness in the organizational context and the workplace type. In Case 1 (Intervention group 1 and 2), the intervention was a part of the organizational processes and structures, as the organization’s senior managers initiated it. The organization was also involved in the overall intervention planning, and its representatives participated in the intervention workshops. Thus, the senior managers and other representatives learned the implementation methodology, received information about the implementation’s progress, and thus had an opportunity to support the teams. In Case 2 (Intervention group 3 and 4), the teams participated without any organizational embeddedness.

All participating units are public organizations funded by tax. Case 1 related to elderly care and disability care, and Case 2 related to health care and social care more broadly (e.g., primary care, rehabilitation, and public health, as well as elderly care and disability care). The staff members in the elderly and disability care group generally had lower education levels than those in health care (e.g., nursing assistants in Case 1 vs. registered nurses and physicians in Case 2). Elderly and disability care workers often also have limited Swedish-language skills, but in other aspects of health care, good knowledge of Swedish is a legal requirement. Table 1 summarizes Case 1 and Case 2.

**Table 1:** Description of the intervention cases including the intervention groups

	Case 1		Case 2	
	*Intervention group* 1	*Intervention group* 2	*Intervention group* 3	*Intervention group* 4
Teams (individual participants)	10 (55)	10 (48)	8 (32)	7 (24)
Type of organization	Elderly and disability care	Elderly and disability care	Health and social care	Health and social care
Time of participation	Feb - May 2016	Aug - Nov 2016	Feb - April 2017	Sept - Nov 2017
Embedded in context	Yes	Yes	No	No

### Intervention development

The intervention development started in autumn 2013. A systematic process using both scientific and practical knowledge was applied. First, a literature review was conducted on the use of training initiatives in implementation science. This search provided information regarding the types of implementation approaches with scientific support, including strategies that were tailored to an organization’s barriers [23], behavioral approaches such as the Behavior Change Wheel framework, and models for the stages of implementation (from exploration to sustainment) [37, 38]. To further understand which components and key competencies are important in facilitating behavioral change in the workplace, the literature search included PsycINFO, PubMed, and Web of Science. The resulting knowledge was combined to form the content and desired outcomes of the intervention. The literature search also provided information about scientifically supported training designs such as applying practical work (experience-based learning), providing opportunities for social interactions, and involving both managers and other professionals [27]. Thus, the intervention delivery and pedagogy were based on the theory of experiential learning [27], the research on training transfer [30], and team learning [8, 19].

Second, this scientific knowledge was supplemented with the views of stakeholders from local health care organizations, as collected through nine interviews in September and October 2013. These interviews were then analyzed using content analysis. The results revealed information about the organizations’ needs, the desired training outcomes in terms of competences, the preferred learning activities, and the contextual circumstances (e.g., the practical factors that influence opportunities to participate in training).

Third, an intervention prototype was developed based on the scientific literature and the interviews. Thereafter, as part of a workshop, national experts (researchers, consultants, and practitioners) in implementation, change management, and health care and social care provided feedback on the prototype.

The intervention was pilot-tested twice (in 2014 and 2015) on a total of 24 teams. Each workshop included systematic evaluations involving questionnaires (296 responses in all). A focus-group interview was also performed with selected participants. The materials were continuously revised, mainly to clarify and simplify them.

### Intervention content

**Fig. 1:**
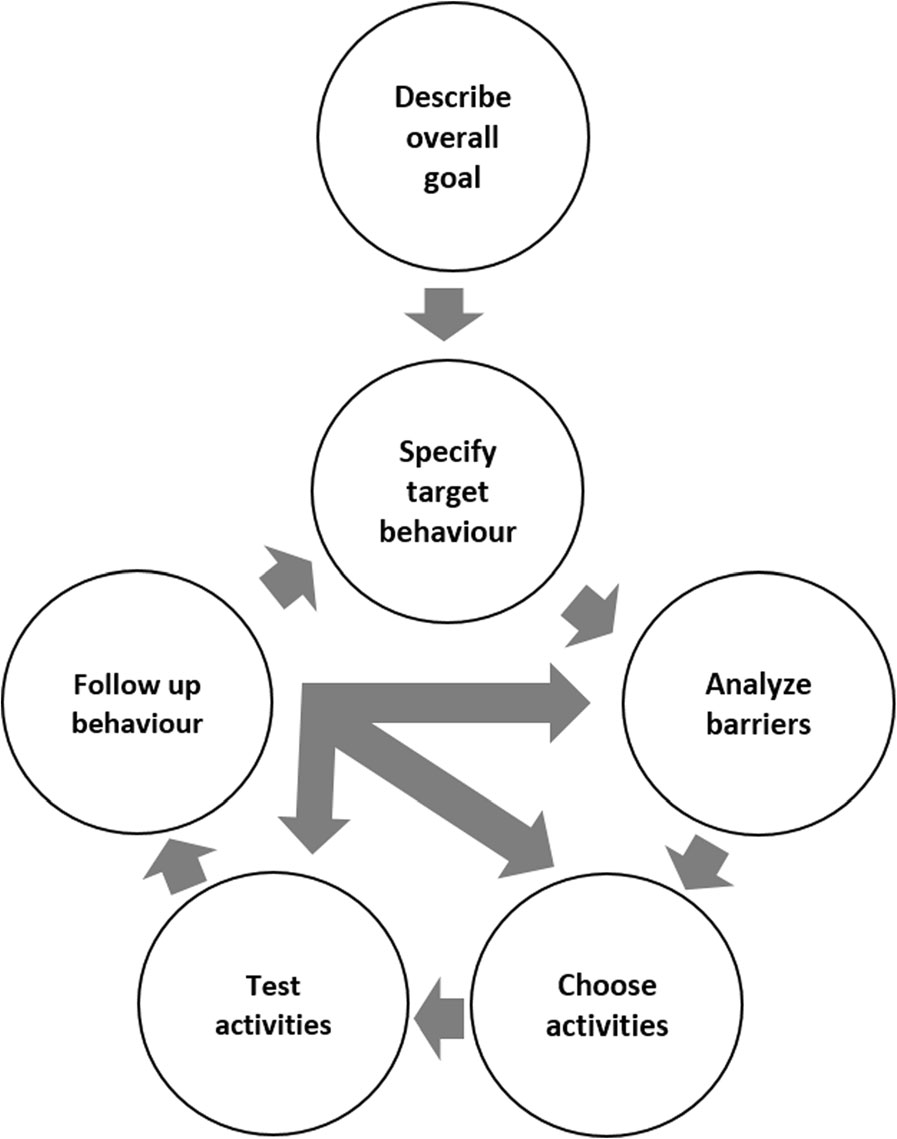
The six step implementation methodology in the BIC intervention

The intervention was delivered in workshops (see Table 2 for the specific content of each workshop). A six-step systematic implementation method (Fig. 1), with exercises and practical work materials, was used. The steps were as follows: 1) describe the current problems and define *the overall goal* of the implementation; 2) list all behaviors that could accomplish that goal, then prioritize and *specify the key (target) behaviors*; 3) systematically *analyze barriers to implementation,* i.e.*, to perform the target behaviors*; 4) *choose implementation activities* that fit the needs identified above; 5) *test the chosen activities in the workplace*; and 6) *follow up* on the implementation’s results to see if the chosen activities have had the desired effect. Thereafter, depending on the results, the process can continue with revised activities, new target behaviors, or a follow-up on the current activities.

**Table 2:** Content of each workshop (for Case 1, the content of workshop 1 was delivered in two workshops)

Workshop 1	Workshop 2	Workshop 3	Workshop 4	Workshop for managers
Introduction to implementation. The implementation methodology - step 1: problems and overall goals	Follow-up on home assignment	Follow-up on home assignment	Follow-up on home assignment	Introduction to leadership
Team work with the implementation case - step 1	Repetition of the implementation methodology	Repetition of the implementation methodology	Team work with the implementation case	Exercise on effective leadership
Exercise on defining goals	Team work with the implementation case - step 2: prioritize behaviors	Introduction to tailored implementation activities	Cross unit feedback on the implementation cases	Introduction to full-range leadership theory
Introduction to behavioral perspective on implementation	Introduction to specifying behaviors	Team work with the implementation case - step 4	Introduction to sustained implementation and handling setbacks	Role play in engaging leadership
Team work with the implementation case - step 2: list behaviors	Team work with the implementation case - step 2: specify target behaviors	Introduction to home assignment: perform step 5 and 6	Team work with the implementation case Countering setbacks, sustainability	Reflection on implementation leadership
Introduction of home assignment: inform colleagues about the gained knowledge	Exercise: barriers to implementation		Lecture on transfer of training	Individual action plan for implementation leadership
	Introduction to barriers to implementation		Cross unit work: apply the implementation methodology to a new case	
	Team work with the implementation case - step 3		Practical exercise: lessons learned	
	Exercise on transfer of training			
	Introduction of home assignment: feedback from colleagues: enablers for the target behaviors

The workshop format combines short lectures, the units’ practical work, peer support (both within and across teams), collaboration between managers and other professionals, between-workshop assignments, feedback from workshop leaders, individual and group reflections, and workshop leaders’ boosting activities (emails, phone calls, and workplace visits). All participating managers were invited to a separate workshop to clarify their role as implementation leaders, following the iLead intervention outline [16, 39].

The delivery format differed somewhat across the cases. In Case 1 (Intervention group 1 and 2), the first workshop was divided into two workshops, as suggested by the organization’s senior managers, to match that organization’s specific conditions (e.g., many staff members with low education levels and lack of fluency in Swedish). Consequently, Case 1 included five half-day workshops, but Case 2 (Intervention group 3 and 4) included four half-day workshops.

### Evaluation

Kirkpatrick’s training-evaluation framework guided the quantitative and qualitative data collection and analysis [36]. This framework differentiates potential effects into four levels: 1) reactions to the training, 2) learning, 3) behavioral changes (i.e., to what degree participants apply what they have learned in practice), and 4) organizational results (Table 3).

**Table 3:** Evaluation levels (Kirkpatrick, 1996 [36]), measured outcomes, data collection methods and time points

Evaluation level	Measured outcome	Data collection methods	Time of data collection
Level 1: Reactions	Participants’ satisfaction with the intervention Participants ‘expectations that the intervention would have positive impact	Questionnaires (paper & pencil)	End of each workshop Post-intervention
Level 2: Learning	Participants’ ratings of their implementation knowledge Participants’ perceptions of their learning	Questionnaires (paper & pencil) Interviews	Baseline and post-intervention Post-intervention
Level 3: Behavior	Participants’ use of the learned implementation steps in practice	Interviews	Post-intervention
Level 4: Results	The impact changed behaviors had on work practices Work units’ readiness for implementation	Interviews Questionnaires (web-based) (all members of the units, including those not participating in intervention)	Post-intervention Case 1*:* Baseline and post-intervention, Case 2: not included

Quantitative and qualitative data were collected to account for the various training effects. The rationale for using mixed methods was to let the methods complement each other and to allow for validation of the findings by triangulation. All intervention participants in each team received an evaluation questionnaire after each workshop (Level 1: reactions), as well as a questionnaire that was given at baseline and after the intervention (Levels 1 and 2: reactions and learning). All participants were also invited to complete an interview to evaluate their learning (Level 2), their behavioral changes (Level 3), and the organizational results (Level 4). For Case 1, all members of the participating units (including professionals and managers who were not participating in the workshops) received web questionnaires at baseline and after the intervention to capture the effects of Level 4. The senior managers involved in Case 1 approved the questionnaire; it was not possible to conduct this step for Case 2. Written informed consent was obtained from all participants.

The interviews were conducted in January and February 2018. Thus, the length of follow-up differed across the four groups. All intervention participants received interview invitations by email and/or phone. Forty-three individuals communicated interest to participate in the interviews, and all of these were selected for the study. Seven interviews were canceled because the participants were unable to attend. This resulted in 35 completed interviews with participants from all four intervention groups. The interviewer (the seventh author) recorded the face-to-face interviews and took detailed notes. One informant declined to be recorded, and three others were interviewed as a group (by their request). The interviews lasted between 15 and 40 min and were performed in Swedish. All the recorded interviews were then transcribed verbatim. Further information on the data collection can be found in Table 4.

**Table 4:** Data collection methods and number of respondents for each intervention group

	Case 1		Case 2	
	*Intervention group 1*	*Intervention* *group 2*	*Intervention group 3*	*Intervention group 4*
Questionnaires to professionals and managers in participating teams	Baseline *n* = 52; Post-intervention *n* = 38	Baseline *n* = 44; Post-intervention *n* = 24	Baseline *n* = 32; Post-intervention *n* = 20	Baseline *n* = 34; Post-intervention *n* = 16
Questionnaires to all professionals working at the units	Baseline *n* = 384 (53%); Post-intervention *n* = 217 (54%)	Baseline *n* = 156 (86%); Post-intervention *n* = 172 (55%)	na	na
Interviews	*n* = 14; 20 months post-intervention	*n* = 11; 14 months post-intervention	n = 3; 6 months post-intervention	*n* = 8; 2 months post-intervention

na = questionnaires not distributed to Case 2

#### Measures

##### Level 1. Reactions

For each workshop, satisfaction with the intervention was measured with two items: relevance and quality [16]. Using Likert scales, the workshop topics were rated from not at all relevant (1) to very relevant (10) and from very low quality (1) to very high quality (10).

Outcome expectancy was measured with two items [41]. A sample item: “I believe that my participation in the intervention has a positive impact on my work.” The response alternatives ranged from strongly disagree (1) to strongly agree (10) on a Likert scale. The groups’ Cronbach’s alpha values varied, α = .76 to .96.

##### Level 2. Learning

A six-item scale for measuring the participants’ implementation knowledge was developed based on a learning scale from another intervention [42]. An example item: “I have enough knowledge to formulate and conduct appropriate activities to support implementation work.” The response alternatives ranged from strongly disagree (1) to strongly agree (10) on a Likert scale. The groups’ Cronbach’s alpha values varied, α = .91 to .93 at baseline and α = .89 to .94 after the intervention.

The participants’ perceptions of their learning were also captured in the interviews via questions about their knowledge regarding the implementation method. (See Additional file 1 for the interview guide.)

##### Level 3. Behavior

The participants’ application of the learned implementation steps was used as a measure of the behavioral changes and was evaluated through interviews questions about their behaviors after the intervention and the impact that the intervention had on their behaviors.

##### Level 4. Results

Interview questions regarding how the implementation was performed and the impact that it had on work practices were used to evaluate the effects at this level. In addition, for Case 1, the work units’ change commitment and change efficacy were measured before and after the intervention via a nine-item scale regarding organizational readiness for implementing change [42]. The wording was altered to make the items easy to understand (e.g., replacing “implementation” with “improvement”). This is a sample item: “In my work group, we are committed to work with improvements.” The response alternatives ranged from strongly disagree (1) to strongly agree (10) on a Likert scale. The Cronbach’s alpha values varied, α = .92 to .95 at baseline and α = .92 to .94 after the intervention.

### Data analyses

Independent-samples t tests were performed to analyze potential baseline differences between the cases and the intervention groups in terms of reactions (Level 1) and learning (Level 2). Paired-samples t tests were performed to analyze whether the intervention groups’ implementation knowledge (Level 2) changed relative to the baseline. To evaluate the organizational results (Level 4) for Intervention group 1 and 2, paired-samples t tests were performed to analyze whether the work units’ readiness for implementation changed relative to the baseline. All analyses were conducted using SPSS 24.

For the qualitative data, the seventh author used the Kirkpatrick training-evaluation framework [36] to conduct a deductive thematic analysis [43]. The author read the transcripts iteratively and focused on the four-level concepts, i.e. reaction, learning, behavior and organizational results, to code the initial data and sorting the different codes into the four levels. The operationalization of the four levels had been defined in a discussion involving all the authors. The third and fourth authors also read the transcripts and discussed the coding with the seventh author.

## Results

### Level 1: reactions

All intervention groups were satisfied with the intervention and had high expectations, believing that it would have a positive impact on their work (Table 5).

**Table 5:** Mean values (standard deviations) for groups in case 1 and 2 for reactions to the intervention (level 1)

	Satisfaction		Expectations of intervention impact
	Relevance of topics^a^	Quality of workshops^a^	Outcome expectancy ^b^
	Mean (SD)	Mean (SD)	Mean (SD)
Case 1			
*Intervention group 1*	8.03 (1.17)	8.13 (1.06)	8.66^**^ (1.42)
*Intervention group 2*	7.80 (1.12)	8.13 (1.10)	7.79^**^ (1.75)
Case 2			
*Intervention group 3*	8.13 (1.02)	7.86 (.95)	7.94 (1.25)
*Intervention group 4*	8.27 (.81)	8.07 (.86)	8.46 (1.46)

^a^= group mean from ratings after each intervention workshop;

^b^= group mean from post-intervention measurement

** = Significant difference between Intervention group 1 and 2: t (62) = 2.46, *p* = .017

### Level 2: learning

The participants’ implementation knowledge increased relative to the baseline in both cases (intervention groups 1–4) (Table 6).

**Table 6:** Mean values (standard deviation) for groups in case 1 and 2 for implementation knowledge (level 2) at baseline and post-intervention

	N	Mean (SD)	t (df)	*p*-value
Case 1				
*Intervention group 1*				
Baseline	52	7.43 (1.60)		
Post-intervention	38	8.53 (.95)	3.29 (37)	.002
*Intervention group 2*				
Baseline	46	7.57 (1.22)		
Post-intervention	24	8.34 (1.10)	3.23 (21)	.004
Case 2				
*Intervention group 3*				
Baseline	65	5.41 (1.46)		
Post-intervention	35	7.82 (.99)	7.00 (18)	.000
*Intervention group 4*				
Baseline	33	5.80 (1.59)		
Post-intervention	16	8.07 (.97)	6.51 (15)	.000

Scale 1–10

The interviews revealed that the participants in both cases learned about the complexity of implementation and became more aware that implementation is time-consuming, as one participant noted:

“It is important not to rush. In thinking about how to work from now on, maybe it is good to let it [the implementation process] take time so that you don’t sit for just half an hour and scribble down a plan ( …) but instead look at it a few times to feel content with the [implementation] plan.” (Case 2)

Participants also stated that the intervention had provided them with a new mind-set regarding implementation. They described how they had learned a structured method for implementation and how the specific parts of the method (e.g., considering implementation in terms of behavioral change) was valuable.

“But I have already gotten into this mind-set. I know that I have to start with certain steps; otherwise, I will not reach the end goal.” (Case 1)

### Level 3: behavior

The participants in both cases reported that they had changed their behaviors by using one or more of the steps that they learned during the intervention. They did not necessarily use all of the steps; rather, they selected those that they perceived most useful in their situations. Few participants reported not using any of the steps, and none reported using all six steps (i.e., the whole implementation process). Some respondents also stated that they planned to use all the steps but had not yet reached the appropriate point in the implementation process to do so.

The first and second steps (“Describe the overall goal” and “Specify a target behavior,” respectively) were the most frequently used steps in both cases. The participants reported that the first step was very useful because it allowed the respondents to think about why the new routine should be implemented and to consider various types of goals when changing a practice, instead of solely focusing on the result of a specific implementation (e.g., putting a guideline into use). Participants reported using the first step as follows:

“I think, in the start, when the problem and goal [are] formulated ( …) is very good and obvious in some way. But I think it’s something we often miss doing; we start thinking in another end. Many times, we start with “What do we want to do?” and then try to motivate why (…) and thus are often too eager to aim for the solution.” (Case 2)

Participants used the second step (“Specify a target behavior”) to obtain clear and tangible behaviors, thereby making the implementation practical and not merely theoretical. They also used this step to establish an agenda (i.e., who does what) and to include all members at the work unit in the implementation. Participants described this step as useful at avoiding a return to past behaviors and described it as follows:

“To specifically concretize—that’s what you do through this method. You concretize exactly what you are going to do: What behavior are we going to change, and what is the overall goal (and to make this clear)? That’s what’s good and maybe even unique about working this way. Otherwise, it often becomes a lot of talk but no action. This [the method] makes it happen.” (Case 2)

The participants in both cases used the third and fourth steps (“Analyze barriers” and “Choose activities,” respectively). They frequently mentioned the motivational function of the implementation strategies. The participants stated that members of their units need to understand why the implementation is necessary and also emphasized the importance of trust and tutorials, as one of the participants noted:

“Maybe to concretize and create some sort of familiarity with why—not why we make changes, but what the purpose of these changes are. That it’s not someone at the municipal town hall that has just decided something that is to be carried out. But that there actually is a purpose.” (Case 2)

Only the participants in Case 1 described the fifth and sixth steps (“Test activities” and “Follow-up behaviors”). They explained that, before the intervention, they had trouble maintaining new behaviors and would often fall back into previous routines. After the intervention, most participants stated that they needed to more actively maintain new routines, as it took some time before they could sustain an implementation in practice. They highlighted the value of continuous follow-up behaviors:

“And the repetition, to go back and follow up, follow up, and follow up. ( …) I personally follow up and ensure that there are checklists that are easy to follow up. Have you signed it, have you not, and why? And to keep reminding about following up.” (Case 1)

Furthermore, in Case 1, only those in authority positions (e.g., the unit managers and others in supervisory roles) applied the steps. The subordinates (e.g., the nursing assistants) usually were not involved in the implementation planning and thus were not in a position to directly use the steps. Instead, they reported changes in their behaviors when their supervisors introduced new routines. By contrast, Case 2 included examples of both managers and other professionals applying the steps.

### Level 4: organizational results

Work units’ readiness for implementation as measured in Case 1 increased relative to the baseline for Intervention group 1, whereas no differences were observed for units in Intervention group 2 (Table 7).

**Table 7:** Mean values (standard deviation) for group 1 and 2 in Case 1 for readiness for implementation (level 4) at baseline and post-intervention

	Case 1			
	*Intervention group 1*		*Intervention group 2*	
	N	Mean (SD)	N	Mean (SD)
Baseline	202	3.79 (.83)	117	3.77 (.64)
Post-intervention	135	4.27 (.62)	95	3.79 (.76)

Scale 1–10

Significant difference from baseline to post-intervention for Intervention group 1:

M_diff_ = .44, SE = 0.07, t = 6.18, *p* < .001)

Some of the interview respondents in Case 1 reported changing their everyday routines as a result of the learned skills. These changes could have positive effects on both the professionals and their clients. For example, in one unit, improving its clients’ meals led to more enjoyment and less distress among the clients. This improvement to the meals even spread to other units in the organization. The participants in Case 2 did not report any examples of learned behaviors influencing their practice. They stated that it was too early for such examples, as their implementation cases from the intervention were still in the planning phase.

## Discussion

Positive effects regarding the two first levels of Kirkpatrick’s evaluation model [36] were found, that is, the participants were satisfied with the intervention and showed improved knowledge and skills relative to the baseline. Some positive changes also occurred for Level 3 (behaviors), even though none of the participating units had systematically applied all the six implementation steps. Regarding Level 4 (organizational results), in Case 1, the work units’ readiness to implement significantly increased. The interviews revealed positive organizational results (i.e., changes in work practices) for Case 1, but not for Case 2.

The training intervention’s goal was to create good opportunities for the transfer of learned skills into work practice. One factor that influences training transfer—*intervention design* [30, 32]—was carefully considered in the intervention’ development. The content and format followed the recommendations from the literature on experience-based learning and training transfer [30]. Training was provided to teams rather than to individuals; the teams’ managers participated; and the participants’ practical experiences were combined with their theoretical knowledge, social interactions, reflections, and peer support [30–32]. Despite these efforts, challenges remained with regard to transferring the learned skills into applied behaviors. This suggests a need to more carefully consider the other two factors that influence training transfer: the organizational context and the participants’ characteristics.

However, influencing the organizational context can be tricky, as it is primarily affected by an organization’s processes and structures, rather than by researchers or intervention developers. Impacting this context requires partnerships between interventionists and organizational stakeholders, such as providing clear indications regarding the impacts of the context changes and the actions that the organization can take. Case 1 included some characteristics of this type of partnership, and some contextual factors were considered in the intervention design (e.g., how a participant’s limited language skills would be handled). In a similar manner, this intervention involved changes in the organizational context to improve transfer (e.g., senior managers following up on the units’ implementation processes). Furthermore, in Case 1, the intervention also affected the organizational context by increasing readiness for implementation. Taken together, the results still suggest that the measures taken to consider the organizational context were not sufficient. The robust evidence regarding the importance of local context to implementation suggests that the organizations themselves need to make sure that the context is receptive for transfer of training [40, 44, 45]. The irony is that the parts of the current intervention in which the units were specifically asked to consider their local context (and make necessary changes) were the parts that the participating units did not undertake. This illustrates how difficult it is to establish changes in an organizational context within a time-limited training intervention and suggests that, to obtain results, researchers need to spend more time and effort understanding the crucial impact of context.

Training interventionists have seldom considered the third component of training transfer—the *participants’ characteristics* (e.g., their motivations and skills). In this intervention, the managers chose the professionals who were involved in the implementation case, but those professionals’ personal features were not specified. Some authors [19] have suggested that individual features should be considered and that the training participants should be carefully selected. Based on the results of this study, we concur with those recommendations. For instance, in Case 1, some participants were actually not expected to directly apply the skills that they had learned in their work practices. To understand this, consider that the selection of participants in a training is commonly based on the general assumptions that competence development is good for all staff members and that everyone should receive an equal amount of training [46]. The practical implications are 1) that the individuals who are best suited to a specific implementation case should be selected to participate and 2) that the organizational routines for defining how these individuals use their acquired knowledge at work should be defined before the training.

A theme that is related to the individual characteristics is the functioning of the selected team members during the training. During training, work teams’ existing group dynamics (e.g., conflicts and power structures) are often present. Conflict related to these dynamics can have a detrimental impact on the team’s learning. In the current intervention, the teams rated the quality of their teamwork after each workshop. The goal was to identify which teams would need additional support during the intervention to establish effective work routines. To improve the design of training interventions, group dynamics should be assessed before selecting the individuals on the team (i.e., before the intervention starts). Some teams could benefit from adding a supplementary intervention [39] to the main intervention so as to improve the effects of the implementation training.

### Methodological considerations

One strength of this study was that the participating units represented distinct settings and worked with diverse implementation cases. This variety strengthens the external validity of the findings. The internal validity was strengthened by the mixed-methods evaluation design, which allowed for data triangulation. Nevertheless, this study’s limitations should be considered when interpreting its results. First, the evaluation was based on self-reported data. Participants’ behavioral changes were evaluated through interviews, which implies a risk that the participants adapted their responses to ensure social desirability [47]. To mitigate this risk, the interviewer was not involved in the intervention. The respondents provided examples of the problems that they experienced when applying what they had learned in the intervention, which suggests that they felt comfortable expressing more than just socially desired answers. Nevertheless, observation would have been a more objective and valid way of evaluating behavioral changes. However, due to the many participating units and this method’s time and cost requirements, observations could not be applied in this study. This study lacked a control group, which made it difficult to determine the magnitude of the changes or to identify whether the changes were solely due to the intervention. To overcome this, we aimed to use a stepped-wedge design; however, for practical reasons, this method was not fully adhered to. Furthermore, as is always the case in evaluations, the chosen time points may have influenced the results, as effects occur on different points in time. The interviews occurred at the same time point even though the groups and cases participated in the intervention at different time points. This may explain why Case 2 showed no evidence of organizational results (Level 4), which takes the longest time to occur. Another explanation may be related to the difference in methods used to assess organizational results in the two cases. In Case 1, questionnaire data from employees, other than the participants in the intervention, showed positive organizational results after the intervention. This data was not possible to collect from employees in Case 2. Thus, it is possible that some organizational results were not detected.

## Conclusions

Although the intervention revealed positive effects in terms of the participants’ satisfaction and knowledge, it also indicated mixed effects in the implementation behaviors and organizational results. These findings suggest that, when designing an intervention to build teams’ implementation capacity, researchers should consider not only the design of the intervention but also the organizational context and the participants’ characteristics, as this maximizes the chances for a successful transfer of learned skills into behaviors.

## Additional file

Additional file 1: Interview guide. The interview guide used in the study (DOCX 18 kb)

## Abbreviations

BIC: Building implementation capacity
R&D: Research and development
