# Supplementary material for: Building implementation capacity (BIC): a longitudinal mixed methods evaluation of a team intervention
Source: BMC Health Serv Res. 2019 May 7;19:287. doi: 10.1186/s12913-019-4086-1 (PMC6505288; doi:10.1186/s12913-019-4086-1)
Supplement: Supplementary file 1 — Interview guide. The interview guide used in the study (DOCX 18 kb) [file 12913_2019_4086_MOESM1_ESM.docx]

Additional file 1. Interview guide

**Introduction:** Can you describe your role at the workplace?

**In what way do you use the knowledge you gained during the intervention (i.e., the steps)?**

Which steps have you used?, Have your learning resulted in anything that you had not expected?

**Have the work methods (i.e., the steps) from the intervention been disseminated to the employees who did not attend the training?**
Some parts that were easier/more difficult to disseminate?, How do you perceive working according to the steps in your workgroup?

**How do you perceive the support and interest for working with the steps in your workplace?**
In what way is the learning form the training encouraged?, Is the training outcomes discussed in the workgroup?

**If the gained knowledge (i.e., the steps) has not been used, what are the main reasons?**

How do you work with improvements instead?
What would be needed for you and your workplace to apply the steps in practice?

**Learning**
Do you feel confident in your ability to work with the different steps?
Do you have enough knowledge / experience to work with the steps?
Have your way of thinking about implementation changed since participating in the training?

**Attitudes and motivation**
Do you feel that your workplace is dedicated to implementation work?, In what way?, What are your general thoughts about conducting implementation work in the workplace?, Why did you want to participate in the training intervention?

***Questions to informants that have not participated in the training intervention***

Have you received any information about the training intervention or about the steps? Which steps have you used? What has been difficult/easy?

Have your new knowledge resulted in anything that you had not expected? Do you want/need more information about the steps?

Do you work with implementation work at the moment? In what way? Who is involved? Why have you chosen that method? What is your opinion about implementation work in general and at your workplace?
